# First do no harm: extending the debate on the provision of preventive tamoxifen

**DOI:** 10.1054/bjoc.2001.2125

**Published:** 2001-09-01

**Authors:** B P Will, K M Nobrega, J-M Berthelot, W Flanagan, M C Wolfson, D M Logan, W K Evans

**Affiliations:** The Health Analysis and Modeling Group, Statistics Canada, 24-Q, R.H. Coats Building, Ottawa, Ontario, K1A 0T6, Canada; The Ottawa Regional Cancer Centre, the University of Ottawa and Cancer Care Ontario, 620 University Avenue, Toronto, Ontario, M5G 2L7, Canada

**Keywords:** breast cancer, microsimulation model, prevention, tamoxifen, population health impacts

## Abstract

The Breast Cancer Prevention Trial (BCPT-P-1) demonstrated that tamoxifen could reduce the risk of invasive breast cancer in high-risk women by 49%, but that it could also increase the risk of endometrial cancer, vascular events and cataracts. This paper provides an estimate of the net health impacts of tamoxifen administration on high-risk Canadian women with no prior history of breast cancer. The results of the BCPT-P-1 were incorporated into the breast cancer and other modules of Statistics Canada’s microsimulation POpulation HEalth Model (POHEM). While the main intervention scenario conformed as closely as possible to the eligibility criteria for tamoxifen in the BCPT-P-1 protocol, 3 additional scenarios were simulated. Predicted absolute risks of breast cancer at 5 years of 1.66%, 3.32% and 4.15% were calculated for women 35 to 70 years of age. When the BCPT-P-1 results were incorporated into the simulation model, the analysis suggests no increase in life expectancy in this risk group. Tamoxifen appeared to be beneficial for women with a 5-year predicted risk of 3.32% or greater. The results of these simulations are particularly sensitive to the reduction in mortality observed in the BCPT-P-1, as well as being sensitive to other characteristics of the simulation model. Overall, the analysis raises questions about the use of tamoxifen in otherwise healthy women at high risk of breast cancer. © 2001 Cancer Research Campaign


*British Journal of Cancer* (2001) **85**(9), 1280–1288
© 2001 Cancer Research Campaign
doi: 10.1054/ bjoc.2001.2125, available online at http://www.idealibrary.com on IDEAL

http://www.bjcancer.com

### First do no harm: extending the debate on the provision of preventive tamoxifen

**BP Will[1], KM Nobrega[1], J-M Berthelot[1], W Flanagan[1], MC Wolfson[1], DM Logan[2] and WK Evans[2]**

[1]The Health Analysis and Modeling Group, Statistics Canada, 24-Q, R.H. Coats Building, Ottawa, Ontario, K1A 0T6, Canada,
[2]The Ottawa Regional Cancer Centre, the University of Ottawa and Cancer Care Ontario, 620 University Avenue, Toronto, Ontario, M5G 2L7, Canada

**Summary** The Breast Cancer Prevention Trial (BCPT-P-1) demonstrated that tamoxifen could reduce the risk of invasive breast cancer in high-risk women by 49%, but that it could also increase the risk of endometrial cancer, vascular events and cataracts. This paper provides an estimate of the net health impacts of tamoxifen administration on high-risk Canadian women with no prior history of breast cancer. The results of the BCPT-P-1 were incorporated into the breast cancer and other modules of Statistics Canada's microsimulation POpulation HEalth Model (POHEM). While the main intervention scenario conformed as closely as possible to the eligibility criteria for tamoxifen in the BCPT-P-1 protocol, 3 additional scenarios were simulated. Predicted absolute risks of breast cancer at 5 years of 1.66%, 3.32% and 4.15% were calculated for women 35 to 70 years of age. When the BCPT-P-1 results were incorporated into the simulation model, the analysis suggests no increase in life expectancy in this risk group. Tamoxifen appeared to be beneficial for women with a 5-year predicted risk of 3.32% or greater. The results of these simulations are particularly sensitive to the reduction in mortality observed in the BCPT-P-1, as well as being sensitive to other characteristics of the simulation model. Overall, the analysis raises questions about the use of tamoxifen in otherwise healthy women at high risk of breast cancer. © 2001 Cancer Research Campaign.

**Keywords**: breast cancer, microsimulation model, prevention, tamoxifen, population health impacts

In 1992, the National Surgical Adjuvant Breast and Bowel Project initiated the Breast Cancer Prevention Trial (BCPT-P-1) to determine whether administering tamoxifen for 5 years prevented invasive breast cancer in women at increased risk for that disease. In a preliminary report with average follow-up of 4.5 years, tamoxifen was shown to reduce the overall risk of invasive breast cancer by 49% (Fisher et al, 1998). In October 1998, the United States Food and Drug Administration (FDA) approved the use of tamoxifen to 'reduce the incidence of breast cancer in women at high risk' for this disease (Zeneca, 1998). A careful examination of the trial's results suggests that there were both beneficial and adverse effects from administering 'preventive' tamoxifen.[1] Moreover, there were wide margins of error in the estimates of the beneficial and adverse effects in the study. Given the debate that has surrounded this study (Bruzzi, 1998; Pritchard, 1998; Fisher, 1999; Lippman and Brown 1999; Noe et al, 1999; Radmacher and Simon 2000), it was considered beneficial to estimate the population health impacts of preventive tamoxifen on women cared for in the Canadian health care environment. The objective of our analysis was to identify under which conditions preventive tamoxifen seemed to provide an overall benefit to women and also to identify if there were any situations under which it might be detrimental.

*Received 12 April 2001*
*Revised 10 July 2001*
*Accepted 23 July 2001*

*Correspondence to:* B Phyllis Will

[1]The term preventive tamoxifen is used throughout the manuscript in the same context as was used in the Breast Cancer Prevention Trial. The authors acknowledge that the FDA approval of tamoxifen was as a drug to reduce the incidence of breast cancer in high risk women and *not* as a breast cancer preventive agent.

Disclaimer: The views expressed in this paper are those of the authors and may not represent those of their respective organizations

### METHODS

### The Population Health Model (POHEM)

POHEM is a microanalytic simulation model developed by the Health Analysis and Modeling Group at Statistics Canada, the Canadian government's central statistical agency.

POHEM creates synthetic longitudinal population samples starting with the birth of each individual in the cohort, and dynamically simulates their ageing, including exposures to risk factors, disease onset conditional on risks, treatment, case fatality and costs (Wolfson, 1994; Berthelot et al, 1997). It does this by synthesizing a large sample of complete individual health and socio-economic biographies. The synthesis process respects and draws upon a myriad of detailed empirical observations. To evaluate tamoxifen, we used a birth cohort and when women met the eligibility criteria they were screened into the simulated therapy. The simulation sample size typically used in this analysis was 4 million women, to ensure that the Monte Carlo error was small relative to the model outputs of interest.

POHEM has been used successfully to develop a comprehensive model of the lifetime costs of diagnosing and treating breast cancer in Canada, to reflect current Canadian risk factors, incidence, diagnostic and therapeutic cancer management practices and costs (Will et al, 1999, 2000). Recently, the model has been modified to evaluate the health and economic impact of interventions such as reduced length of in-hospital stay for breast cancer surgery and post-mastectomy locoregional radiotherapy for node-positive Stage II breast cancer patients (Will et al, 1998; Evans et al, 2000).

For this analysis, tamoxifen's impact on breast and endometrial cancer (BC and EC), deep vein thrombosis (DVT), stroke, coronary heart disease (CHD), fractures and cataracts has been evaluated. In addition, mortality from causes other than those listed above was explicitly modeled, but without any preceding morbidity. A wide variety of data sources were culled (references are contained in the original breast cancer reports – Will et al, 1999; Will et al, 2000). Breast and endometrial cancer incidence data were obtained from the national cancer registry by age group. Breast cancer risk factors were taken from the National Breast Screening Study and from vital statistics records. Baseline incidence for the remaining diseases under study was calculated from the electronic health care records maintained by the province of Manitoba.

Mortality rates for the individual diseases were modelled to reflect as closely as possible those from Canadian vital statistics records. No mortality was associated with cataracts or DVT in the model, but they have still been modeled because their incidence is significantly affected by tamoxifen. The use of hormone replacement therapy (HRT) is included since it affects whether women are eligible for tamoxifen. These modules have been validated by ensuring that the incidence (when known), overall life expectancy, and disease-specific mortality rates generated by POHEM correspond to those observed in Canada. See Appendix A for details on the diseases in the model and the methodology used.

### Assumptions in modeling preventive tamoxifen for Canadian women

POHEM was used to simulate the administration of preventive tamoxifen to a representative cohort of Canadian women. The reference case assumed no provision of preventive tamoxifen. The main intervention scenario (Scenario 1) conformed as closely as possible to the eligibility criteria for tamoxifen in the BCPT-P-1 protocol. Women were defined to be at increased risk for breast cancer in the simulation if they:

- were 60 to 70[2] years of age; or
- were 35–59 years of age with a 5-year predicted risk of breast cancer of at least 1.66%; or
- had a history of lobular carcinoma *in situ* (LCIS);
- had no history of deep vein thrombosis or endometrial cancer; and
- had not taken hormone replacement therapy in the 3 months prior to starting on tamoxifen.

### Estimating the 5-year predicted risk

For this analysis, we used the Gail algorithm for estimating the probability (risk) of breast cancer over time (Gail et al, 1999) in the same way as in the BCPT-P-1 to predict each simulated woman's 5-year risk of breast cancer. POHEM's simulated individual risk profiles, in aggregate, replicate Canadian risk factor distributions for age, family history, nulliparity or age at first live birth, number of breast biopsies, and age at menarche. For each synthetic woman, the odds ratios from Gail et al were used in combination with the age-specific Canadian breast cancer incidence rates to estimate the 5-year predicted risk. If this predicted risk was high enough to place the woman in the eligible range, the intervention simulated was 20 mg of tamoxifen per day for 5 years. Tamoxifen administration was assumed to have been stopped at the onset of deep vein thrombosis, breast cancer, or endometrial cancer.

### Incorporating all outcomes from the BCPT-P-1

When the results of a clinical trial are reported, much emphasis is put on the primary endpoint for which it was designed. In the case of preventive tamoxifen, the highlight of the trial was the 49% reduction in breast cancer incidence. However, other outcomes were measured (e.g., endometrial cancer, deep vein thrombosis, etc.) with varying degrees of precision. In performing an evaluation of the global impact of an intervention, many researchers use the point estimates of the different outcomes. However, since some outcomes are not statistically significant, or are of borderline statistical significance, some judgement is required as to which are likely to be real. This means that different researchers could arrive at different conclusions regarding which outcomes to include. Instead of using the point estimates of the relative risks (which would force a subjective decision about which of the relative risks are significant), we used the entire information on their distribution, as published from the BCPT-P-1.

Table 1 summarizes the relative risks (RRs) of developing certain diseases, and their confidence intervals, as derived from the BCPT-P-1. Our approach is to perform a multivariate analysis that takes into account parametric uncertainty based on the distribution of the input parameters. POHEM draws from the distribution of the input parameters and associates distinct parameter values to different sub-samples. For this analysis, 40 sub-samples were used. Effectively, this is similar to conducting ‘pseudo-trials’ that would have resulted in point estimates within the confidence interval for each of the outcomes under study. The variation between sub-samples is used to calculate standard errors for the simulation results. In this manner, the multivariate distribution of the outcomes can be estimated and parametric uncertainty can be incorporated into the simulation run. Furthermore, using the information from the distributions allows for the calculation of standard errors that reflect the uncertainties of the outcome. For example, if a relative risk has a very wide confidence interval (i.e. its effect is highly uncertain), it will not have any significant impact on the final results. Using the approach of ‘pseudo-trials’ allows us to keep the information on all outcomes measured in the trial without having to make subjective decisions.

### The long-term effects of tamoxifen

Since the median follow-up period in the BCPT-P-1 trial was less than 5 years, the longer-term consequences of tamoxifen use in women without breast cancer are not known. It was assumed that the relative risks (RRs) for breast and endometrial cancer and fractures would return (linearly) to 1.0 within 5 years following cessation of therapy. It was also assumed that the RRs of coronary heart disease would return to 1.0, one year after cessation of therapy. For the other outcomes, it was assumed that the RRs would return to 1.0 immediately after cessation of therapy, as some of the biological effects of tamoxifen are thought to be promptly reversed on cessation of the drug.

[2]Even though the trial did not specify a maximum eligibility age, we assumed that, in the Canadian context, it would be questionable to offer preventive tamoxifen to women over age 70. This is the only criterion that differs from those in the BCPT-P-1.

*© 2001 Cancer Research Campaign*

**Table 1** Summary of results of the BCPT-P1

| Disease incidence | Age group | RR | (CI) |
|---|---|---|---|
| Breast cancer | 35–49 | 0.56 | (0.37, 0.85) |
| | 50–59 | 0.49 | (0.29, 0.81) |
| | 60+ | 0.45 | (0.27, 0.74) |
| Endometrial cancer | 35–49 | 1.21 | (0.41, 3.60) |
| | 50+ | 4.01 | (1.70, 10.90) |
| Coronary heart disease | All | 1.15 | (0.81, 1.64) |
| Hip fractures | All | 0.55 | (0.25, 1.15) |
| Spine fractures* | All | 0.74 | (0.41, 1.32) |
| Wrist fractures* | All | 0.93 | (0.69, 1.27) |
| Stroke | 35–49 | 0.76 | (0.11, 4.49) |
| | 50+ | 1.75 | (0.98, 3.20) |
| Deep vein thrombosis* | 35–49 | 1.39 | (0.51, 3.99) |
| | 50+ | 1.71 | (0.85, 3.58) |
| Cataract onset* | All | 1.14 | (1.01, 1.29) |
| Cataract surgically treated* | All | 1.57 | (1.16, 2.14) |
| **Mortality** | | | |
| Other Cancer Mortality** | All | 0.57 | (0.31, 0.97) |
| Other Non-Cancer Mortality** | All | 1.21 | (0.69, 2.22) |

RR = relative risk; CI = confidence intervals.
*assumed non-fatal.
**RR for mortality is assumed to be 1 in base scenarios.
***RRs and CIs are from Tables 3, 4, 6, 7, 8, 9 and 11 of Fisher et al (1998) reference.
Note:
The RR for 'Other Cancer Mortality' is the ratio of the number of deaths due to cancer (excluding breast and endometrial) in the intervention group divided by the number of person-years of follow-up for the intervention group over the same quantity from the control group. Source: Table 11 of Fisher et al (1998) reference.
The RR for 'Other Non-Cancer Mortality' is the ratio of the number of deaths due to causes other than cancer, coronary heart disease, hip fracture or stroke in the intervention group divided by the number of person-years of follow-up for the intervention group over the same quantity from the control group. Source: Table 11 of Fisher et al (1998) reference.

### Reference case and new scenarios

The reference case assumes that no tamoxifen was administered. Scenario 1 simulates the BCPT-P-1. Since tamoxifen is not administered without the potential for harmful side effects, 3 additional scenarios were simulated in order to evaluate the effectiveness of preventive tamoxifen on different sub-populations. The second scenario used more conservative eligibility criteria. It was assumed that women were at high risk if they were 35 to 70 years of age with a 5-year predicted risk of ≥ 1.66%, as calculated by the Gail model. The last two scenarios assumed an even more restricted population of women for preventive tamoxifen. Tamoxifen was administered in these scenarios if the woman was between 35 and 70 and had a 5-year predicted risk of breast cancer of at least 3.32% (twice the eligibility criteria of BCPT-P-1), and 4.15%, respectively. A range of additional scenarios has been modelled (≥ 2.08%, ≥ 2.49%, ≥ 2.91%, ≥ 3.74%), but is not presented. The results are available on request.

### Sensitivity analyses

Sensitivity analyses are used to determine the impact on the results of changes to one or more parameter assumptions in the analysis. If the conclusions drawn from a simulation model are not affected by such sensitivity analyses, it can be assumed that the conclusions are robust with regard to the assumptions examined.

One sensitivity analysis explored the impact of a longer duration of the protective effects of tamoxifen for cancer after cessation of therapy, tailing off over a 10-year, rather than a 5-year period. In this sensitivity analysis, we also determined the impact of alternatively assuming that the RRs of cancers and fractures would return (linearly) to 1.0 within 10 (rather than 5) years following cessation of therapy. A second analysis retained the baseline assumption of a 5-year tailing off of protective effects of tamoxifen, but instead, assumed that tamoxifen had a beneficial effect on mortality from cancers other than breast and endometrial (RR of 0.57), and a detrimental effect on all other causes of death (RR of 1.21), as inferred from Table 11 of the BCPT-P-1 study report.

In this latter case, our analysis of the detailed counts of deaths by cause suggests a statistically significant beneficial effect on 'other cancer' mortality (i.e. excluding breast and endometrial cancer), even though the published results showed no significant difference in overall mortality between the placebo and tamoxifen arms of the trial, and the study found no significant difference in 'other cancer' incidence between the 2 arms. This result appeared paradoxical to us. These effects on mortality by cause may be due to the relatively small numbers of events and the short follow-up time for mortality effects. Nonetheless, a sensitivity analysis was performed to determine if the published reduction in other cancer mortality and increase in other non-cancer mortality might have an impact on the overall results.

### RESULTS

Since each POHEM scenario covers a different population with a different underlying risk of breast cancer and other diseases, the reference for each scenario is provided in Table 2. Table 3 provides the changes observed between each 'reference case without tamoxifen' and the preventive tamoxifen scenario. Table 4 presents the sensitivity analyses of the results to alternative assumptions regarding the long-term effects of tamoxifen.

In the reference case (Scenario 1 in Table 2), life expectancy for eligible Canadian women in 1991 was 83.9 years, and 67.8% of all women could expect to survive to age 80. Almost 9% could expect to have breast cancer at some point in their lives, and to spend almost 11 years living with the disease, and 3.2% could expect to die from breast cancer.

When we look cross-sectionally at women in the year 2000, using BCPT-P-1 eligibility criteria, 23% of the Canadian population would be eligible for tamoxifen. However, from a population health perspective, it is also important to look at the lifetime potential of being eligible for this drug. Overall, applying the BCPT-P-1 eligibility criteria to Canadian women (Scenario 1 in Table 2) would result in over 85% of all women being subjected to tamoxifen administration at some point in their lives. These women taking tamoxifen could anticipate a significant decrease ($P < 0.05$) in life expectancy of about 0.04 years, while the proportion surviving to age 80 would decrease by about 0.2%. The burden of breast cancer would fall, but the burdens of CHD, endometrial cancer, stroke, hip fracture and cataracts would all increase. Mortality from other diseases in the simulation would also fall. These simulation results suggest that preventive tamoxifen may not be beneficial to the health of Canadian women when offered according to the eligibility criteria of BCPT-P-1.

To determine if tamoxifen might be beneficial to various subsets of women at higher risk of breast cancer, several alternatives were

 *© 2001 Cancer Research Campaign*

**Table 2** Base case outcomes for each scenario

| Eligibility criteria | Scenario 1 BCPT-P-1 | Scenario 2 5-year BC risk ≥ 1.66, age 35–70 | Scenario 3 5-year BC risk ≥ 3.32, age 35–70 | Scenario 4 5-year BC risk ≥ 4.15, age 35–70 |
|---|---|---|---|---|
| Lifetime percentage of women eligible | 85.1 | 42.3 | 4.0 | 1.7 |
| Life expectancy (years) | 83.9 | 83.9 | 84.3 | 84.4 |
| Percentage alive at age 80 | 67.8 | 68.0 | 69.2 | 69.2 |
| BC Incidence (%) | 8.7 | 11.1 | 16.4 | 18.6 |
| BC Mortality (%) | 3.2 | 4.1 | 5.9 | 6.5 |
| Average person-years with BC | 10.4 | 10.8 | 10.1 | 9.8 |
| CHD incidence (%) | 49.2 | 48.5 | 47.1 | 46.9 |
| CHD mortality (%) | 23.3 | 23.1 | 22.9 | 22.9 |
| EC incidence (%) | 1.8 | 1.8 | 1.6 | 1.5 |
| EC mortality (%) | 0.5 | 0.4 | 0.4 | 0.4 |
| Stroke incidence (%) | 32.7 | 32.5 | 32.5 | 32.3 |
| Stroke mortality (%) | 7.9 | 7.9 | 8.0 | 7.9 |
| Hip fracture incidence (%) | 18.9 | 18.8 | 18.5 | 18.5 |
| Hip fracture mortality (%) | 3.3 | 3.3 | 3.4 | 3.4 |
| Cataract incidence (%) | 30.8 | 29.7 | 28.3 | 27.9 |
| Other cancer mortality (%) | 13.7 | 13.5 | 12.8 | 12.6 |
| Other cause mortality (%) | 44.6 | 44.4 | 43.7 | 43.5 |

BC = breast cancer; EC = endometrial cancer; CHD = coronary heart disease.

**Table 3** Impacts of alternative scenarios for administering tamoxifen

| Eligibility criteria for each scenario | Change under alternative scenarios | | | |
|---|---|---|---|---|
| | Scenario 1 Similar to BCPT-P-1 | Scenario 2 5-year BC risk ≥ 1.66, age 35–70 | Scenario 3 5-year BC risk ≥ 3.32, age 35–70 | Scenario 4 5-year BC risk ≥ 4.15, age 35–70 |
| Life expectancy (LE) (years) | –0.04 | –0.01$^{NS}$ | 0.06 | 0.07 |
| Probability of increase in LE (%) | 30.0 | 43.0 | 75.0 | 75.0 |
| Percentage alive at age 80 | –0.2 | –0.1$^{NS}$ | 0.2 | 0.4 |
| BC incidence (%) | –1.2 | –1.4 | –2.5 | –3.1 |
| BC mortality (%) | –0.5 | –0.7 | –1.1 | –1.3 |
| Average person–years with BC | –0.69 | –0.6 | –0.60 | –0.69 |
| CHD incidence (%) | 0.3 | 0.4 | 0.7 | 0.8 |
| CHD mortality (%) | 0.2 | 0.2 | 0.3 | 0.5 |
| EC incidence (%) | 1.4 | 1.4 | 1.5 | 1.6 |
| EC mortality (%) | 0.4 | 0.4 | 0.4 | 0.4 |
| Stroke incidence (%) | 0.2 | 0.4 | 0.9 | 0.9 |
| Stroke mortality (%) | 0.1 | 0.1 | 0.2 | 0.3 |
| Hip fracture incidence (%) | 0.3 | 0.1$^{NS}$ | –0.1$^{NS}$ | –0.5 |
| Hip fracture mortality (%) | 0.1 | 0.0$^{NS}$ | –0.1$^{NS}$ | –0.1 |
| Cataract incidence (%) | 0.3 | 0.4 | 0.7 | 0.6 |
| Other cancer mortality (%) | 0.0$^{NS}$ | 0.0$^{NS}$ | 0.0$^{NS}$ | 0.0$^{NS}$ |
| Other cause mortality (%) | –0.1 | 0.0$^{NS}$ | 0.2 | 0.2 |

Note: The changes are relative to the appropriate reference column in Table 2.
BC = breast cancer; EC = endometrial cancer; CHD = coronary heart disease.
NS = Not significant at $P \leq 0.05$ (otherwise, the change is significant).

simulated. In the first alternative, eligibility for tamoxifen after age 60 was made more stringent by requiring a 5-year predicted risk of at least 1.66% for all women 35 to 70 years of age. The baseline characteristics and the results of this simulation are shown as Scenario 2 in Tables 2 and 3, respectively. Under this scenario, 42% of women would be eligible for preventive tamoxifen at some point in their lives. This population would have a higher risk of breast cancer than that in the first scenario. For this subpopulation, there would be a decrease in life expectancy of 0.01 years, which is not statistically significant. In this simulation, the increases in mortality from CHD, endometrial cancer, stroke and hip fracture would counterbalance any benefit of tamoxifen in reducing breast cancer mortality.

Scenario 3 shows the impact of preventive tamoxifen therapy for women aged 35 to 70, who, based on the Gail algorithm, would have a 5-year predicted risk of breast cancer of 3.32%. For this

*© 2001 Cancer Research Campaign*

**Table 4** Sensitivity analysis of estimated impacts of tamoxifen for alternative risk scenarios

| | **BCPT-P1** | **10 year return to baseline risks after stopping tamoxifen** | **Other cancer mortality RR = 0.57, Other non-cancer mortality RR = 1.21** |
|---|---|---|---|
| Percentage of all women ever administered tamoxifen | 85.1 | 85.1 | 85.1 |
| Life expectancy (years) | –0.04 | –0.03 | 0.13 |
| Percentage alive at age 80 | –0.2 | –0.1 | 0.5 |
| BC Incidence (%) | –1.2 | –1.5 | –1.1 |
| BC Mortality (%) | –0.5 | –0.7 | –0.5 |
| Average person–years with BC | –0.69 | –0.83 | –0.69 |
| CHD incidence (%) | 0.3 | 0.4 | 0.7 |
| CHD mortality (%) | 0.2 | 0.2 | 0.4 |
| Endometrial cancer incidence (%) | 1.4 | 1.9 | 1.5 |
| Endometrial cancer mortality (%) | 0.4 | 0.6 | 0.4 |
| Stroke incidence (%) | 0.2 | 0.2 | 0.4 |
| Stroke mortality (%) | 0.1 | 0.1 | 0.1 |
| Hip fracture incidence (%) | 0.3 | $0.1^{NS}$ | 0.4 |
| Hip fracture mortality (%) | 0.1 | $0.0^{NS}$ | 0.1 |
| Cataract incidence (%) | 0.3 | 0.3 | 0.6 |
| Other cancer mortality (%) | $0.0^{NS}$ | $0.0^{NS}$ | –0.7 |
| Other cause mortality (%) | –0.1 | –0.1 | 0.5 |

BC = breast cancer; CHD = coronary heart disease.
NS = Not significant at $P \leq 0.05$ (otherwise, the change is significant).

sub-population, there would be a significant increase ($P < 0.05$) in life expectancy of 0.06 years, and a 0.2% increase in the proportion reaching age 80.

Finally, Scenario 4 shows the results of administering tamoxifen to the 1.7% of the population of women whose risk of breast cancer would, at some point in their life course, be 2 1/2 times higher than that in the BCPT-P-1, at 4.15%. This scenario estimates a significant increase ($P < 0.05$) in life expectancy of 0.07 years. For this population, there would be a marked decrease (3.1%), in the incidence of breast cancer and a decrease of 0.7 years with breast cancer for this sub-population.

Table 4 shows the results of 2 sensitivity analyses, juxtaposed against the 'standard' BCPT-P-1 intervention scenario (Scenario 1). The first of these analyses explores the impact of a longer duration of anti-cancer protective effects from tamoxifen, with the benefit tailing off over a 10-year, rather than a 5-year period. Even when it is assumed that the effects of tamoxifen last over a 10-year period following cessation of therapy, the life expectancy of women would not increase. The results are consequently not sensitive to this assumption. The second analysis retains the baseline assumption of a 5-year tailing off of the protective effects of tamoxifen, but assumes that tamoxifen has a beneficial effect on mortality from cancers other than breast and endometrial (RR of 0.57), and a detrimental effect on all other causes of death (RR of 1.21). The simulation results indicate an increase in life expectancy of 0.13 years accompanied by an increase in the proportion reaching age 80. In this scenario, the probability of an increase in life expectancy was estimated to be 60%. When compared to the reference case scenario, it can be seen that the results of the simulation are highly sensitive to the assumption that there is no reduction in other cancer mortality.

### DISCUSSION

Tamoxifen has been used as an adjuvant therapy for metastatic breast cancer and to decrease the incidence of contralateral breast cancer for over 2 decades (Jordan, 1990, 1995; Love et al, 1991; Early Breast Cancer Trialists' Collaborative Group, 1992; Tomas et al, 1995; Fisher et al, 1996; Early Breast Cancer Trialists' Collaborative Group, 1998). This has created considerable debate (Bruzzi, 1998; Pritchard, 1998; Fisher, 1999; Lippman and Brown 1999; Noe et al, 1999; Radmacher and Simon, 2000), particularly regarding issues such as impact on cardiovascular disease (Love et al, 1991), duration of administration (Jordan, 1990; Fisher et al, 1996) and quality of life (Day et al, 1999). However, there is a general consensus that, for breast cancer patients, the survival benefits of tamoxifen far outweigh the adverse effects (Jordan, 1990; Early Breast Cancer Trialists' Collaborative Group, 1998).

More recently, attention has been focused on tamoxifen's potential to prevent breast cancer in 'high risk' women. The release of the findings of the Breast Cancer Prevention Trial (BCPT-P-1) in March 1998 resulted in unprecedented media coverage and precipitated additional debate regarding the risks and benefits of tamoxifen administration. The trial showed a 49% reduction in breast cancer, but also showed that there were some life-threatening adverse effects associated with tamoxifen administration (Gail et al, 1999). Adding to the debate were the results of two European tamoxifen chemoprevention trials, which were unable to confirm the P-1 trial findings, but which also used different sample sizes and eligibility criteria (Powles et al, 1998; Veronesi et al, 1998).

One of the major issues concerning the BCPT-P-1 results is that the trial population has not been followed long enough to produce

 *© 2001 Cancer Research Campaign*

reliable mortality data or to determine the net health benefit to society of a tamoxifen breast cancer prevention strategy, (Pritchard (1998) and Lippman and Brown (1999)) (See April 19, 2000 JNCI for comments / critiques by Rockhill et al, and responses by Lippman and Brown, and by Fisher).

Gail and his colleagues have developed a methodology to determine the population of women most likely to benefit from preventive tamoxifen (Gail et al, 1999), and the FDA in the United States has given approval for the use of tamoxifen for women at increased risk of breast cancer. However, according to some, there is still uncertainty as to whether tamoxifen only delays the appearance of breast cancer or truly prevents the disease itself (Zeneca, 1998; Radmacher and Simon, 2000).

Should *all* healthy women 60 years of age and older take tamoxifen, when only 8.3% are likely to get breast cancer after that age? Given that the BCPT-P-1 showed a 49% reduction in incidence of invasive breast cancer for women on the tamoxifen arm, how many of these have actually been prevented permanently, how much of the reduction is due to the inhibition of growth of occult tumours, and what impact will there be on life time breast cancer mortality? Assuming that there is support for the thesis that tamoxifen inhibits the growth and progression of ER-positive tumours, which are generally found in older women, what proportion of premenopausal women at high risk would actually benefit from its administration? There has also been considerable discussion regarding the importance of evaluating breast cancer risk, as well as the methodology for doing so (Jordan, 1990; Costantino et al, 1999; Gail et al, 1999; Radmacher and Simon, 2000; Smith and Hillner, 2000). Finally, concern has been expressed over the ethics of administering tamoxifen to a healthy population of women and about the importance of considering its clinical toxicology (Jordan, 1990, 1995; Emanuel et al, 2000).

In our model, the eligibility criteria, risk factors and relative risks from the BCPT-P-1 were applied to a simulated cohort of Canadian women, using Canadian incidence and mortality rates and breast cancer management patterns. Besides breast cancer, the analysis also assessed tamoxifen's effects on endometrial cancer, CHD, DVT, stroke, hip fractures, cataracts, and all other causes of mortality.

Certain important differences between our analysis and that of other researchers are relevant. The most important difference is that the POHEM microsimulation includes life expectancy, and not just the more proximate end-points of breast cancer incidence or prevalence (Fisher et al, 1998; Radmacher and Simon, 2000). We evaluated the lifetime impact and net benefit of providing preventive tamoxifen to high-risk women for a 5-year period, whereas others used 5-year risks or effects (Gail et al, 1999; Smith and Hillner, 2000). Additionally, Noe and colleagues used incidence rates from the trial, rather than the baseline incidence in an actual population. In our analysis, we considered the overall impact of all diseases mentioned in the BCPT-P-1 trial, whereas Noe et al (1999) based their analysis on only those diseases showing statistically significant differences (breast and endometrial cancer, pulmonary embolism and cataract surgery).

Although Gail et al developed tools to address the harmful risks of preventive tamoxifen, their risk–benefit analysis was based upon the number of events. In POHEM, life-years gained or lost due to these events are also considered. In Gail's analysis, endometrial cancer, pulmonary embolism, hip fracture, stroke and breast cancer were all considered to be of equal weight. By looking directly at the mortality associated with these events, one can more accurately assess the impact of these events on lifetime health. Smith and Hillner (2000) have stated that tamoxifen for breast cancer prevention should be cost-effective under nearly all circumstances, but acknowledge that the risk reduction due to tamoxifen might not result in a reduction in breast cancer deaths. However, since their analysis did not take into account the life-years lost due to the harmful side effects of tamoxifen, they may have over-estimated the benefits of tamoxifen. Furthermore, the POHEM approach incorporates the uncertainty associated with the input parameters as measured by the BCPT-P-1, allowing for the calculation of confidence intervals.

When comparing the results of clinical trials to 'real-life' situations, it is important to distinguish between efficacy and effectiveness. The BCPT-P-1 trial showed the efficacy of administering tamoxifen in a clinical trial setting to reduce breast cancer incidence (Fisher et al, 1998). However, practice patterns, tests, follow-up, and survival within a clinical trial setting are not the same as in the general population. In order to assess effectiveness, the setting of the analysis should be the general population, with standard practice patterns and outcomes. For this reason, in our POHEM simulation, standard disease progression and mortality data were used. In the case of endometrial cancer, there were no deaths in the BCPT-P-1. Although most endometrial cancers might be prevented with proper screening and tests, in Canada (NCIC), as in the United States (SEER) (Ries et al, 1997), there is still mortality associated with this cancer. It has recently been reported that endometrial cancers discovered in women taking adjuvant tamoxifen are more advanced at diagnosis and less likely to have a favourable outcome compared with those in women who have not taken tamoxifen (Bergman et al, 2000).

Our study has several limitations. First, the results are based on hypothetical rather than real cases, and the proportion of cases receiving specific tests and treatments is based upon the proportion of cases in various categories in the databases used. Survival data were taken from administrative sources. However, even with these limitations, the breast cancer model of diagnostic and therapeutic procedures and disease progression has been calibrated by reproducing incidence and overall life expectancy, and approximating disease-specific mortality, as seen in Canada.

Few women develop breast cancer in their lifetime. However, according to the criteria of the BCPT-P-1, 85% of women would be eligible to take preventive tamoxifen at some point in their lives. Based on the results of this POHEM simulation, although tamoxifen has a substantial benefit in reducing breast cancer incidence and mortality, the detrimental effects of tamoxifen on endometrial cancer, coronary heart disease, stroke, and deep vein thrombosis may counter-balance the protective effect tamoxifen has on breast cancer for the majority of the women meeting the eligibility criteria of BCPT-P-1. As a consequence, the results of this simulation analysis raise important questions about the use of preventive tamoxifen. In the United States, tamoxifen is approved to reduce the incidence of breast cancer in high risk women who are 35 or older and have a 5-year predicted risk of $\geq 1.67\%$, as calculated by the Gail model (Gail et al, 1999).

The results of our simulations are highly sensitive to the assumption regarding 'other cancer' mortality. If it is assumed that the reduction in mortality for 'other cancers' observed in the BCPT-P-1 is not artefactual, our analysis suggests that preventive tamoxifen could be effective even for the BCPT-P-1 entry criteria. However, this mortality reduction is somewhat paradoxical. As there was no difference in the incidence of other cancers in the two

*© 2001 Cancer Research Campaign* 

arms of the trial (RR = 1.0), a reduction in mortality would imply that tamoxifen had a therapeutic effect on these cancers. Alternatively the difference could be an artefact of the short follow-up.

As a consequence of these uncertainties, additional trials and longer follow-up of prevention trials would be useful to determine whether preventive tamoxifen can reduce all-cause mortality, as well as the more proximate endpoint of breast cancer incidence. New oestrogen-suppressing drugs, such as raloxifene and anastrozele are now being introduced and evaluated, and will require the same kind of careful evaluation given to tamoxifen. The ongoing Multiple Outcomes of Raloxifene Evaluation (MORE) (Cummings et al, 1998) and the Study of Tamoxifen and Raloxifene (STAR) (National Cancer Institute, 1999) clinical trials are attempts to find an intervention that will prevent breast cancer with a minimum of side effects. Overall, the analysis raises questions about the use of preventive tamoxifen in otherwise healthy women at high risk of breast cancer.

### ACKNOWLEDGEMENTS

The authors gratefully acknowledge the important contributions of Dr Eva Tomiak, Dr Shailendra Verma and Christel Le Petit, who were instrumental in the development of the original breast cancer model. In addition, we would like to thank Dr Michael Fung Kee Fung for contributing his expertise in surgical oncology. Finally, we would like to thank Rolande Belanger and Brenda Moore for their administrative assistance.

### REFERENCES

Bergman L, Beelan MLR, Gallee MPW, Hollema H, Benraadt J, Leeuwen FE and the Comprehensive Cancer Centres' ALERT Group (2000) Risk and prognosis of endometrial cancer after tamoxifen for breast cancer. *Lancet* **356**: 881–887

Berthelot J-M, Le Petit C and Flanagan W (1997) *Use of longitudinal data in health policy simulation models*, American Statistical Association Proceedings, California: 120–129

Bruzzi P (1998) Tamoxifen for the prevention of breast cancer. *BMJ* (Editorial) **316**: 1181–1182

Costantino JP, Gail MH, Pee D, Anderson S, Redmond CK, Benichou J and Wieand HSI (1999) Validation studies for models projecting the risk of invasive and total breast cancer incidence. *J Natl Cancer Inst* **91(18)**: 1541–1548

Cummings SR, Norton L and Eckert S (1998) Raloxifene reduces risk of breast cancer and may decrease the risk of endometrial cancer in postmenopausal women. Two-year findings from the Multiple Outcomes of Raloxifene Evaluation (MORE) trial. *Proc Am Soc Clin Oncol* **17:2a** (abstr 3)

Day R, Ganz PA, Costantino JP, Cronin WM, Wickerham DL and Fisher B (1999) Health-related quality of life and tamoxifen in breast cancer prevention: a report from the National Surgical Adjuvant Breast and Bowel Project P-1 Study. *J Natl Cancer Inst* **17(9)**: 2659–2669

Early Breast Cancer Trialists' Collaborative Group (1992) Systemic treatment of early breast cancer by hormonal, cytotoxic, or immune therapy. *Lancet* **339(8785)**: 71–85

Early Breast Cancer Trialists' Collaborative Group (1998) Tamoxifen for early breast cancer: an overview of the randomised trials. *Lancet* **351**: 1451–1467

Emanuel EJ, Wendler D and Grady C (2000) What makes clinical research ethical? *JAMA* **283(20)**: 2701–2711

Evans WK, Will BP, Berthelot J-MB, Logan DM, Mirsky DJ and Kelly N (2000) Breast cancer: better care for less cost: is it possible? *Int J Technol Assess Health Care* **16(4)**: 1168–1178

Fisher B (1999) National Surgical Adjuvant Breast and Bowel Project Breast Cancer Prevention trial: A reflective commentary. *J Natl Cancer Inst* **17(5)**: 632–639

Fisher B, Dignam J, Bryant J, DeCillis A, Wickerham DL, Wolmark N, Costantino J, Redmond C, Fisher ER, Bowman DM, Deschênes L, Dimitrov NV, Margolose RG, Robidoux A, Shibata H, Terz J, Paterson AHG, Feldman MI, Farrar W, Evans J and Lickley HL (1996) Five versus more than five years of tamoxifen therapy for breast cancer patients with negative lymph nodes and estrogen receptor-positive tumors. *J Natl Cancer Inst* **88(21)**: 1529–1541

Fisher B, Costantino J, Wickerham DL, Redmond CK, Kavanah M, Cronin WM, Vogel V, Robidoux A, Dimitrov N, Atkins J, Daly M, Wieand S, Tn-Chiu E, Ford L, Wolmark N and other National Surgical Adjuvant Breast and Bowel Project Investigators (1998) Tamoxifen for prevention of breast cancer: report of the national surgical adjuvant breast and bowel project P-1 study. *J Natl Cancer Inst* **90(18)**: 1371–1388

Fisher B (2000) Correspondence *J Natl Cancer Inst* **92(8)**: 659

Gail MH, Costantino JP, Bryant J, Croyle R, Freedman L, Helzlsoeur K and Vogel V (1999) Weighing the risks and benefits of tamoxifen treatment for treating breast cancer. *J Natl Cancer Inst* **91(21)**: 1829–1846

Jordan VC (1990) Tamoxifen for the prevention of breast cancer. In: De Vita VT, Hellman S, and Rosenberg SA, editors. Cancer Prevention. Philadelphia (PA): Lippincott; 1–12

Jordan VC (1995) Tamoxifen: toxicities and drug resistance during the treatment and prevention of breast cancer. *Annu Rev Pharmacol Toxicol* **35**: 195–211

Lippman SM and Brown PH (1999) Tamoxifen prevention of breast cancer: an instance of the fingerpost. *J Natl Cancer Inst* **91(21)**: 1809–1818, Lippmann SM and Brown PH (2000) Correspondence *JNCI* **92(8)**: 658

Love RR, Wiebe DA, Feyzi JM, Newcomb PA and Chappell RJ (1991) Effects of tamoxifen on cardiovascular risk factors in postmenopausal women. *Ann Intern Med* **115**: 860–864

National Cancer Institute May 25, 1999 Press release regarding the Study of Tamoxifen and Raloxifene (STAR) trial. *http://cancertrials.nci.nih.gov/NCI CANCER TRIALS/zones/TrialInfo/News/star/starpr.asp*

Noe LL, Becker RV III, Gradishar WJ, Gore M and Trotter JP (1999) The cost effectiveness of tamoxifen in the prevention of breast cancer. *Am J Manag Care* **5(6)**: S389–406

Powles T, Eeles R, Ashley S, Easton D, Chang J, Dowsett M, Tidy A, Viggers J and Davey J (1998) Interim analysis of the incidence of breast cancer in the Royal Marsden Hospital tamoxifen randomised chemoprevention trial. *Lancet* **352**: 98–101

Pritchard K (1998) Is tamoxifen effective in prevention of breast cancer? *Lancet* Commentary **352**: 80–81

Radmacher MD and Simon R (2000) Estimation of tamoxifen's efficacy for preventing the formation and growth of breast tumors. *J Natl Cancer Inst* **92(1)**: 48–53

Ries LAG, Kosary CL, Hankey BF, Miller BA, Harras A and Edwards BK, editors (1997) SEER Cancer Statistics Review: 1973–1994, National Cancer Institute. NIH. Pub No. 97–2789. Bethesda, MD

Rockhill B, Colditz G and Kaye J (2000) Comment. *J Natl Cancer Inst* **92(8)**: 657–658

Smith TJ and Hillner BE (2000) Tamoxifen should be cost-effective in reducing breast cancer risk in high-risk women. *J Clin Oncology* **18(2)**: 284–286

Tomas E, Kauppila A, Blanco G, Apaja-Sarkkinen M and Laatikainen T (1995) Comparison between the effects of tamoxifen and toremifene on the uterus in postmenopausal breast cancer patients. *Gynecol Oncol* **59(2)**: 261–266

Veronesi U, Maisonneuve P, Costa A, Sacchini V, Maltoni C, Robertson C, Rotmensz N and Boyle P (1998) Prevention of breast cancer with tamoxifen: preliminary findings from the Italian randomised trial among hysterectomised women. *Lancet* **352**: 93–97

Will BP, Berthelot J-M, Houle C, Tomiak EM, Verma S and Evans WK (June 1998) The economic impact of locoregional radiotherapy (LRRT) on all post-surgical stage II breast cancer patients in Canada. Poster presentation – International Society for Technology Assessment in Health Care – Ottawa

Will BP, Le Petit C, Berthelot J-M, Tomiak EM, Verma S and Evans WK (1999) Diagnostic and therapeutic approaches for non-metastatic breast cancer in Canada and their associated costs. *Br J Cancer* **(9/10)**: 1428–1436

Will BP, Berthelot J-M, Le Petit C, Tomiak EM, Verma S and Evans WK (2000) Estimates of the lifetime costs of breast cancer treatment in Canada. *Eur J Cancer* **36**: 724–735

Wolfson MC (1994) POHEM – a framework for understanding and modelling the health of human populations. *Wld Hlth Statist Quart* **47**: 157–176

Zeneca DE (1998) Tamoxifen prescribing information (package insert.) Wilmington

### APPENDIX A

The following paragraphs provide brief overviews of the data sources and information used to build the major disease modules in POHEM, including those developed specifically for the analysis of the impact of preventive tamoxifen on the health of Canadian

 *© 2001 Cancer Research Campaign*

women. For all diseases mentioned below, relative risks (RRs) from the BCPT-P-1 were applied to the respective incidence rates.

### Breast cancer module

The sources of data for the POHEM breast cancer module have previously been described in detail (Will et al, 1999; Will et al, 2000). The breast cancer module starts with age-gender incidence patterns based on the Canadian Cancer Registry (only female breast cancer is modeled) (National Cancer Institute of Canada, 1995). For this analysis, the average incidence rates are adjusted for risk factor exposures, using relative risks from the Gail model. The risk factors for the breast cancer tamoxifen intervention are derived from the following sources: the (Canadian) National Breast Screening Study (NBSS) provided information on family history of breast cancer, age at menarche and age at menopause (Miller et al, 1992); Vital Statistics provided data on age of the mother at the birth of a first child and nulliparity; hormone replacement therapy (HRT) was derived from health surveys; and the number of previous breast biopsies was calculated from the Manitoba electronic database of health care records (Roos et al, 1987; Roos, 1999).

Since breast cancer survival critically depends on staging, data on stage at diagnosis were obtained through special arrangements with provincial cancer registries. The stage distribution used for the model was Stage I–46%, Stage II–41%, Stage III–7%, and Stage IV–6%.

Following diagnosis of initial treatment, breast cancer can progress along different paths. Based on the stage of the disease at the time of diagnosis, treatment approaches and follow-up schedules are assigned according to observed proportions, as part of the Monte-Carlo microsimulation method. For women diagnosed at Stage I, II or III, three transitions are possible – from diagnosis to local recurrence, from diagnosis to distant recurrence (or metastasis), or directly from diagnosis to death.

Once a woman has a local recurrence, transitions to distant recurrence or to death are possible. Finally, when a woman is diagnosed with a distant recurrence (Stage IV), the recurrence is assigned to one of two sites: visceral or non-visceral. The only transition allowed at this point is to death, and that transition occurs at a different pace depending on the site. In general, the visceral site has a poorer survival.

Durations between these various discrete events or survival times have been estimated from detailed longitudinal microdata obtained from Saskatchewan and Northern Alberta. These stochastic waiting times are typically represented by piecewise Weibull distributions.

### Coronary heart disease module

The progression of coronary heart disease (CHD) is based on Weinstein et al (1987), with case fatality matching 1991 Canadian CHD mortality from vital statistics. Incidence of CHD is modeled as one of four possible events: sudden death, cardiac arrest, myocardial infarction or angina. Because no national CHD incidence rates are available in Canada, baseline incidence rates are derived by inverting the Weinstein disease progression model, working back from 1991 Canadian CHD mortality rates, while taking account of the underlying risk factor distribution, and Framingham relative risk functions (from section 37 of the Framingham reference study) (Abbott, 1987). The major risk factors for CHD (cholesterol, blood pressure, smoking, age and gender) are derived from the cross-sectional 1978–1979 Canada Health Survey, (Health and Welfare Canada, 1981) and are smoothed using a transport flow analysis to provide the simulation of longitudinal risk behaviours (Gentleman et al, 1990).

### Hormone replacement therapy (HRT)

HRT use was one of the eligibility criteria of the BCPT. The incidence of HRT usage has been derived from the cross-sectional prevalence of HRT in Canada’s 1994 National Population Health Survey, and a small survey on duration of use conducted by the University of Ottawa, standardized to the general population of women.

The relative risks of hip fracture, CHD and breast cancer are all affected by HRT. The magnitudes of these effects have been taken from the Office of Technology Assessment (OTA) study of Hormone Replacement Therapy (U.S. Congress, 1995) and a literature review. The risk of CHD is assumed to instantly decrease by half when HRT is taken and to return to normal levels when HRT is stopped.

### Hip fracture

The hip fracture model (Flanagan et al, 1997) is based on the natural history of bone mineral density (BMD) which has been extracted from the U.S. Office of Technology Assessment Study of HRT. Baseline hip fracture rates are derived from the 1986–1990 Manitoba electronic physician and hospitalization records.

### Endometrial cancer

The Canadian Cancer Registry was used to obtain endometrial cancer incidence by age groups (National Cancer Institute of Canada, 1995). Although there was no mortality associated with endometrial cancer within the BCPT-P-1 trial, the mortality rates for endometrial cancer and the other individual diseases were modeled to reflect those from Canadian vital statistics records.

### Stroke

Electronic health care records from the province of Manitoba were used to calculate incidence and survival for stroke. The time till death due to stroke was calculated as a piecewise Weibull function.

### Cataracts

Cataracts were also modeled in several stages, using the Manitoba database referred to above. Once a woman was diagnosed with cataracts, the time until surgery on the first eye was modeled using a piecewise Weibull function. The time between the first and second eye surgery was also modeled based on a Weibull curve.

### Deep vein thrombosis (DVT)

Incidence of DVT was modeled using information from the Manitoba database referred to above. No mortality was modeled for DVT.

© 2001 Cancer Research Campaign 

### Parametric uncertainty

To reflect parametric uncertainty in our simulation, 40 replicates of each scenario were simulated. For each replicate, a vector of RRs was drawn using a Latin hypercube sample design (Ma et al, 1993; Cronin et al, 1998) from independent lognormal distributions with the 95% confidence intervals shown in Table 2. Then, 40 cohorts of 100 000 women each were simulated, based on these 40 vectors of RRs, for a total of 4 000 000 cases. The variability of several of the key outcomes was then derived from their distributions over the 40 replicates. From these sub-populations, the probability of a positive life expectancy can be estimated empirically.

### REFERENCES

Abbott RD and McGee D (1987) The Framingham Study: an epidemiological investigation of cardiovascular disease. Section 37: The probability of developing certain cardiovascular diseases in eight years at specified values of some characteristics. U.S. Department of Health and Human Services, NIH Publication 87–2284

Cronin K, Legler JM and Etzioni R (1998) Assessing uncertainty in microsimulation modelling with application to cancer screening interventions. *Statist Med* **17**: 2509–2523

Flanagan W, Berthelot J-MB and Le Petit C (1997) Should long-term hormone replacement therapy in postmenopausal women be promoted? Conference Proceedings, Canadian Health Economics Research Association

Gentleman J, Robertson D and Tomiak M (1990) Smoothing procedures for simulated longitudinal microdata. Analytical Studies Branch Research Paper Series Number 32, Ottawa, Statistics Canada

Health and Welfare Canada (1981) The health of Canadians: report of the Canada Health Survey. Statistics Canada, Catalogue 82–538E

Ma J, Ackerman E and Yang J-J (1993) Parameter sensitivity of a model of viral epidemics simulated with Monte Carlo techniques. 1. Illness attack rates. *Int J Biomed Comput* **32**: 237–253

Miller AB, Baines CJ, To T, and Wall C (1992) Canadian National Breast Screening Study 1: Breast cancer detection and death rates among women aged 40 to 49 years. *Can Med Assoc J* **147**: 1459–1476

National Cancer Institute of Canada (1995) Canadian Cancer Statistics 1995. Toronto, Canada

Roos LL, Nicol JP and Cageorge SM (1987) Using administrative data for longitudinal research: comparisons with primary data collection. *Journal of Chronic Diseases* **40(1)**: 41–49

Roos LL and Nicol JP (1999) A research registry: uses, development, and accuracy. *J Clin Epidemiol* **52**: 39–47

U.S. Congress, Office of Technology Assessment (1995) Effectiveness and costs of osteoporosis screening and hormone replacement therapy, Washington DC, U.S. Government Printing Office, 1995–387–789: 47402

Weinstein MC, Coxson PG, Williams LW, Pass TM, Stason WB and Goldman L (1987) Forecasting coronary heart disease incidence, mortality, and cost: The Coronary Heart Disease Policy Model. *Am J Public Health* **77(11)**: 1417–1426

Will BP, Le Petit C, Berthelot J-M, Tomiak EM, Verma S and Evans WK (1999) Diagnostic and therapeutic approaches for non-metastatic breast cancer in Canada and their associated costs. *Br J Cancer* **(9/10)**: 1428–1436

Will BP, Berthelot J-M, Le Petit C, Tomiak EM, Verma S and Evans WK (2000) Estimates of the lifetime costs of breast cancer treatment in Canada. *Eur J Cancer* **36**: 724–735

 *© 2001 Cancer Research Campaign*
